# Erosion–Corrosion Failure Analysis of a Mild Steel Nozzle Pipe in Water–Sand Flow

**DOI:** 10.3390/ma16227084

**Published:** 2023-11-08

**Authors:** Rehan Khan, Michał Wieczorowski, Darko Damjanović, Mohammad Rezaul Karim, Ibrahim A. Alnaser

**Affiliations:** 1 Department of Mechanical Engineering, College of Electrical and Mechanical Engineering, National University of Sciences and Technology, Islamabad 44000, Pakistan; 2 Faculty of Mechanical Engineering, Institute of Applied Mechanics, Poznan University of Technology, 3 Piotrowo St., 60-965 Poznan, Poland; michal.wieczorowski@put.poznan.pl; 3 Mechanical Engineering Faculty in Slavonski Brod, University of Slavonski Brod, Trg Ivane Brlić Mažuranić 2, 35 000 Slavonski Brod, Croatia; ddamjanovic@unisb.hr; 4 Mechanical Engineering Department, College of Engineering, King Saud University, Riyadh 11421, Saudi Arabia

**Keywords:** pipe nozzle, corrosion, erosion, CFD

## Abstract

Several leaks appeared in a mild steel (MS) pipe jet nozzle installed in a direct impact test rig after a few months of operation in erosive flow at the Centre for Erosion–Corrosion Research. The locations of perforation leaks were primarily upstream, but severe wall thinning was also noticed adjacent to the exit section. In this paper, a failure analysis was carried out on the leaking of a pipe jet nozzle, and the results are discussed in detail. The investigation carried out includes visual observation, scanning electron microscopy, 3D scanning, energy-dispersive spectroscopy, and laser profilometry measurements. In addition, numerical simulations based on computational fluid dynamics (CFD) and the discrete phase model (DPM) were conducted to investigate the root cause of the failure of leaks in the pipe jet nozzle. Further CFD-DPM simulations were performed on three different pipe jet designs for liquid–solid flow conditions, and were compared to find an alternative design to prevent the failure of the pipe jet nozzles. It was found that the increase in turbulence along with multiple impacts of particles on the wall generate leaks and cracks in the pipe jet nozzle. Moreover, the CFD-DPM showed a five-fold reduction in the maximum erosion rate; this was observed in the replacement of failed pipes with the proposed alternative nozzle pipe design featuring a chamfer reducer section. The CFD-DPM analysis of all geometric configurations showed that alteration of reducer section design has the greatest impact on erosive wear mitigation.

## 1. Introduction

A pipe jet nozzle is used to develop sufficient flow and momentum of fluid in a round jet configuration that can sustain fluid emerging at high speed across long distances [1]. Erosion and corrosion are the primary root causes of the failure of pipe fittings [2,3,4,5,6]. In carbon steel pitting, cracking, and corrosion attacks are the main causes of failure. Systematic failure analyses of pipe components help to mitigate failure and suggest directions to improve design configurations and operating conditions to prevent such failures in the future [7,8,9].

Leaks were found at locations adjacent to the inlet at the top of a Mild Steel (MS) pipe jet nozzle installed in a direct impact test rig for the testing of components in hydroabrasive flow conditions after a few months of operation in multiphase flow conditions at the Centre for Erosion–Corrosion Research, NUST, Pakistan. Visual inspection shows the presence of cracks and one leak location in the upstream location, and severe wall thinning was observed in the downstream section. Another small leak perforation was noticed on the inlet slightly shifted from the top of the pipe. Based on our analysis, the pipe jet nozzle made from mild steel (MS), which is prone to failure due to erosion–corrosion after operation for a short period; leaks were detected within 4 months of the operation.

In the literature, several studies on the erosion–corrosion failure of pipe components under industrial operating conditions have been conducted [10,11,12]. Yan et al. [13] investigated the occurrence of leakage in elbow pipes during service at sewage stripping units. Failure analysis was carried out via qualitative and quantitative techniques and computational fluid dynamics (CFD) simulation. They found that dissolved corrosive medium in sewage and uneven distribution of flow velocity are the root causes of elbow pipe failure. Meng et al. [14] evaluated the performance degradation of jet pipe servo valves by employing experimental and numerical procedures under the wedge erosion of jet amplifiers. The results show that the dynamic behavior of the jet pipe servo valve will degrade with the extent of the erosion of the wedge. Although many researchers have investigated the primary mechanism of erosive wear, they reported that cutting and pitting are the paramount mechanisms of erosion [15,16]. Predicting pipeline erosive wear can help researchers in the hydrocarbon and mineral processing industries to understand the primary causes of therefore prevent erosion damage; several studies have been conducted regarding erosive wear evaluations of different flow devices such as valves [17,18,19,20], pumps [21,22,23,24], turbines [25,26,27,28] and cyclone separators [29,30,31].

Moreover, some experimental studies on erosion using direct impact test apparatus have been conducted in the literature; unfortunately, there is still a lack of experimental studies on the erosion of nozzle pipes in liquid–solid flow conditions. A limited erosion study involving mitigation of erosion in nozzle pipes has been established to reduce particle–wall impaction (which is very detrimental to pipelines), alongside alternative nozzle design suggestions for erosion prevention and mitigation.

**Figure 1:**
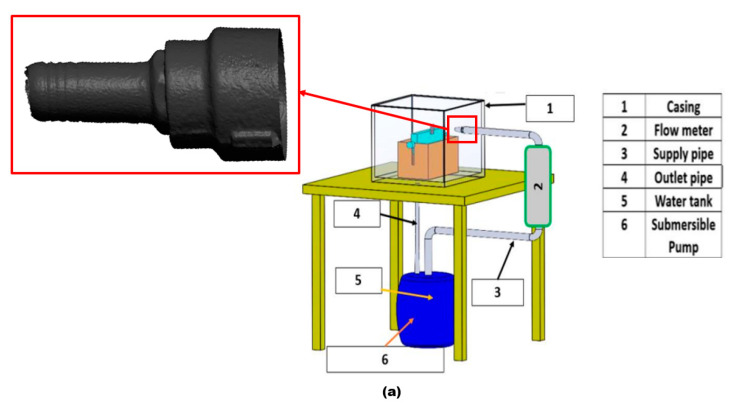

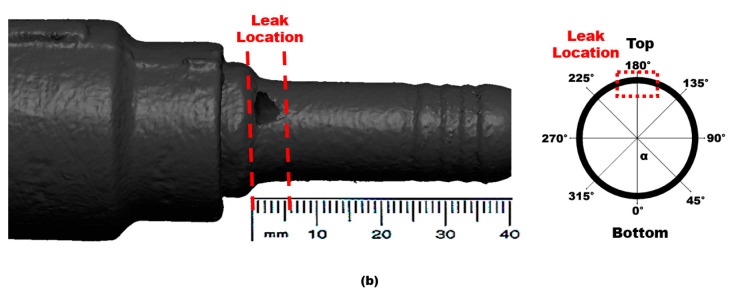
(**a**) Direct impact test rig. (**b**) Location of the leak on jet pipe nozzle.

In this research, a root-cause-of-failure analysis was performed on a jet pipe nozzle connected to a direct impact test rig, as shown in Figure 1. The carrier phase flowing through the pipe jet nozzle is water with sand particles 300 µm in size. The unexpected perforation in the pipe jet nozzle took place after 300 h of operation. Then, the failed nozzle pipe was replaced with a new pipe fitting. The root causes of the failure of the nozzle pipe had to be identified to mitigate financial losses and ensure the safety of the operating process. Therefore, the reason for the failure was determined via qualitative and quantitative analysis. Computational fluid dynamics (CFD), the discrete phase model (DPM), and particle tracking techniques were employed to study the erosion-induced mechanism. Microscopic examination, roughness measurements, and confocal microscopy were used to further understand the underlying process, and related countermeasures were developed to mitigate erosion–corrosion issues. Additionally, CFD-DPM simulations were performed on three different designs of pipe nozzle to find alternative design choices for the replacement of failed nozzle pipes.

## 2. Materials and Methods

The failed jet pipe nozzle material is mild steel, the nozzle pipe diameter is 10 mm, and the pipe wall thickness is 3 mm. The chemical composition of a mild steel pipe nozzle with (wt%) 0.22 C, 0.40 Mn, 0.012 S, <0.21 P, <0.03 Si and <0.4 Cu was obtained from the manufacturer. The carrier fluid contains sand microparticles with an average size of 300 µm. The flow velocity of the slurry is 6 m/s, with a particle concentration of 2 wt%. The nozzle pipe suddenly failed during normal operating conditions after 300 hrs. The leak location is found adjacent to the nozzle inlet at the top of the pipe. A failure analysis was performed, utilizing visual observation, microscopic analysis, thickness measurements, computational fluid dynamics (CFD), and the discrete phase model (DPM). In the current work, the erosion–corrosion behavior of the failed nozzle pipe was evaluated via 3D scanning and SEM-EDS. Besides, using SEM, the surface roughness of the samples was measured using a laser profilometer before further analysis. A portable roughness tester, Mitutoyo SURFTEST SJ-210, was used to measure the mean surface roughness (Ra). The failed nozzle pipe was cut into two sections in such a way that the Ra value was captured at different locations (x = 0, 10, 20, 30, and 40 mm) and circumferential angles (α). After quantification of Ra values, a 3D Olympus DSX1000 confocal microscope was used to obtain a 3D surface profile of the location with the maximum Ra value, which is given in Section 3.1.

### Numerical Setup and Computational Mesh

**Figure 2:**
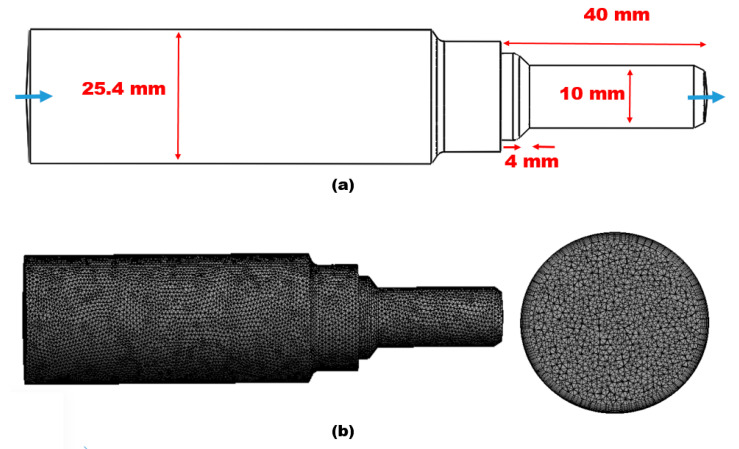
(**a**) Geometrical configuration; (**b**) computational mesh.

The ANSYS fluent CFD-DPM module was used to evaluate the erosion phenomena of the pipe jet nozzle. Computational fluid dynamics (CFD) and the discrete phase model (DPM) were coupled to simulate and track the motion of discrete phases, such as solid particles, in a fluid flow. The DPM uses a Lagrangian approach to track the motion of individual solid particles transported through the carrier fluid. A tetrahedral mesh was used to solve and produce the full flow domain in order to represent the numerical setup, providing reliable numerical simulations and minimizing numerical diffusion errors. As illustrated in Figure 2, a stepwise refining approach was used close to the proximity of walls to better capture the flow pattern.

The Oka [32] erosion prediction model was selected to quantify the erosion rate, and the K-epsilon turbulence model was used to predict the behavior of the particle, which is fluid-flow-dependent. In order to capture the flow field near the wall and increase the calculation accuracy of CFD-DPM, boundary layer grids with 10 layers were set near the wall. To further improve the efficiency of numerical simulation while ensuring the optimal computation cost, verification of grid independence was carried out. The computational mesh used for CFD-DPM is a structured hexahedron with a total number of cells of 460,000; the pipe’s geometrical configuration is shown in Figure 2.

## 3. Results and Discussion

### 3.1. Morphology Analysis

**Figure 3:**
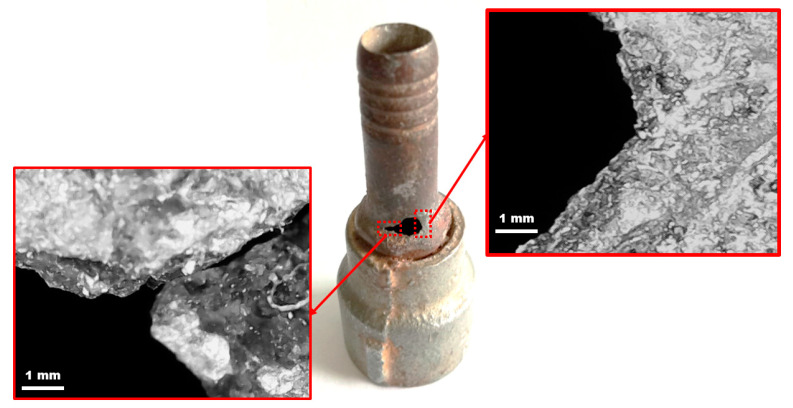
Macrographs of the location of the failed nozzle pipe leak.

Due to long-term exposure to the slurry, the surface of the failed nozzle pipe has serious perforation traces, and the leak is located adjacent to the inlet location. Figure 3 shows the geometry of the failed nozzle pipe connected to the reducer pipe and the location of the leak. The failure was observed at the top at a 180° annular angle adjunct to the inlet of the nozzle pipe. It can be identified that the wall thinning results in the development of cracks and leaks upstream, as shown in Figure 3. The presence of cracks and wall thinning reveals the intense erosion–corrosion of material, and the combined effect of erosive wear and corrosion with high turbulent flow cause the leak perforations.

The macroscopic appearance of elbow leaks is depicted in Figure 3. The top of the pipe close to the inlet is more prone to erosion-corrosion. On the basis of the visual inspection, the localized erosion-corrosion at the pipeline upstream is found and perforated in a spherical shape that extends in the annular direction of the pipe. This kind of failure signifies that corrosion and erosion together caused the leak. The traces of corrosion products inside the pipe indicate that erosion enhances corrosion due to the impaction of particles with carrier fluids. The erosion process is often first exposed to maximal particle impaction and turbulent flow on the pipe surface. These perforation site seen in Figure 3 suggests passive films are destroyed from the upstream section, and then the erosion–corrosion initiates as the carrier fluid impacts the surface.

**Figure 4:**
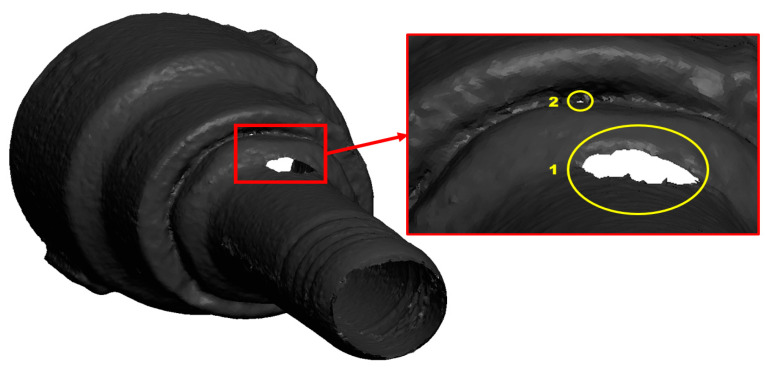
3D scan of leak locations on the nozzle pipe.

A 3D scan of leak morphology in Figure 4 shows that the leak spreads at locations 1 and 2 at the top of the pipe, with the development of sharp corners. The leak perforations at location 2 are remarkably small in diameter and are developed in a rounded shape; the leak at location 1 of the nozzle pipe shows severe degradation due to erosion and corrosion.

**Figure 5:**
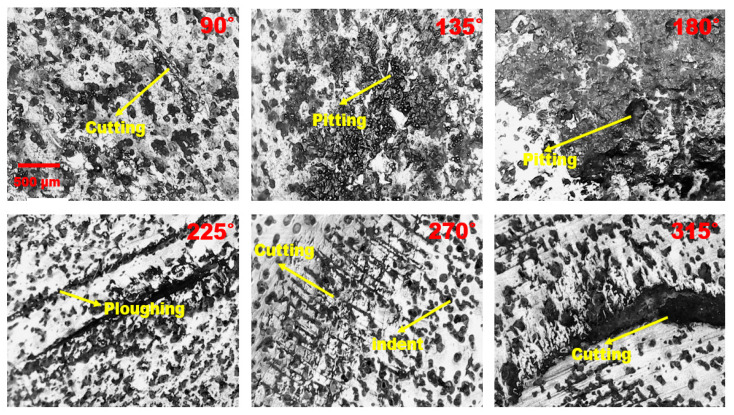
Microscopic images taken from different circumferential angles (α) adjacent to the leak location.

Figure 5 shows microscopic images of erosion–corrosion at different circumferential angles (α) of the nozzle pipe. The corrosion attack originated inside the nozzle and was observed adjacent to the leak according to the microscopic evaluation of the worn surface. Cutting, pitting, and indentations occur primarily on the internal surface, and the severe pitting is concentrated at a circumferential angle of 180° adjacent to the leak location. Ploughing and cutting were observed at a circumferential angle of 225° and 270°. According to the microscopic analysis, the leaks developed as a consequence of the wall thinning phenomenon, which is a particular erosion process caused by the impaction of particles and carrier fluid on the metal surface. Based on Figure 5, the extent of the rusting and the greater number of pits developed due to the acceleration in the corrosion rate at circumferential angles of 90° and 180°.

**Figure 6:**
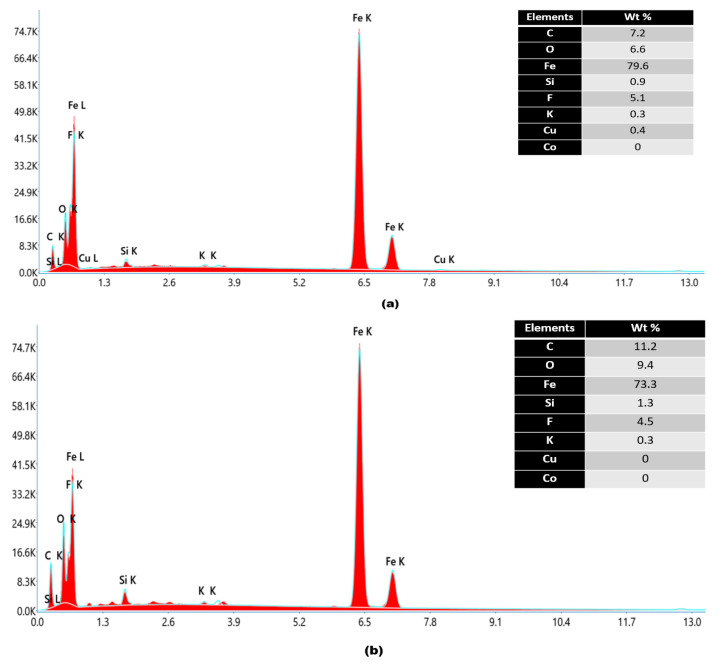
EDX analysis of nozzle pipe (**a**) inlet and (**b**) exit.

Plastic deformation can also cause the breakage of the passive oxide film in the mild steel, increase the surface energy, and further accelerate the erosion–corrosion. Thus, small pits become large leak sites due to severe plastic deformation. According to Figure 5, cutting and ploughing are observable at circumferential angles of 225°, 270°, and 315° after erosion–corrosion. Comparing our results with those of Khan et al. [3], ploughing and cutting are more obvious in the steel pipe when there is a low or medium angle of impaction between the erodent and the wall. EDX was used to examine the elemental composition of the worn surface at the inlet and outlet of the nozzle pipe. The trace of Si indicates sand particles embedded in the surface, and iron atoms’ reduction in the inlet section in comparison with the outlet indicates that an oxidization reaction has taken place, resulting in severe corrosion with the development of scales of Fe_2_O_3_, as shown in Figure 6. During the erosion–corrosion process, the corrosive medium eroded the nozzle’s internal surface, resulting in the development of loose and soft corrosion products. The corrosion products formed on the surface were easily removed due to particle impaction, and in turn, led to serious damage. These locations might become the origin of cracks causing spalling of the worn surface and resulting in leak perforations.

### 3.2. Thickness Measurements

**Figure 7:**
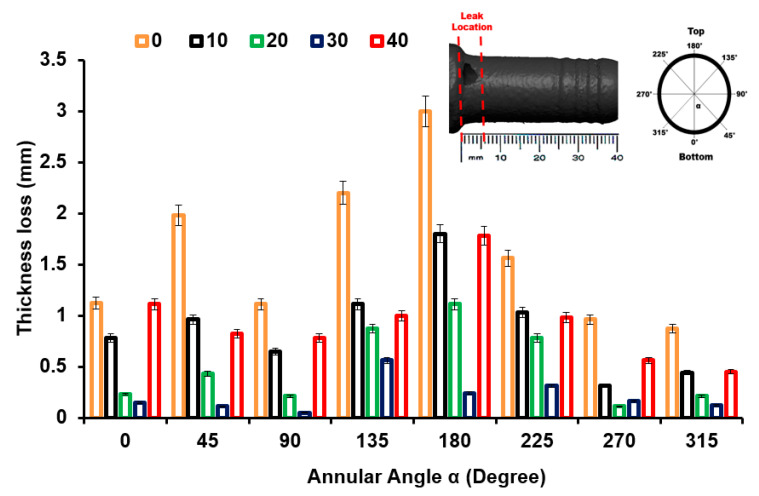
Thickness loss at a different locations in the nozzle pipe.

Thickness at different locations (x = 0, 10, 20 30, and 40 mm) and circumferential angles (α) were quantified by employing a digital micrometer three times at each location (Figure 7). This quantitative method for thickness reduction from upstream to downstream identifies the location of severe wall thinning.

The thickness plotted at different locations and annular angles (α) along the axial length is shown in Figure 7. Surprisingly, the inlet and outlet sections encounter maximum wall thinning. As is shown in Figure 7, the thickness measured at eight circumferential angles corresponds to the positions of erosion and wall thinning. The serious erosion is mainly located at α = 180° and x = 0, and the area with minimum wall thinning is located at α = 270° and x = 20 mm. It was also observed that in comparison with the outlet, the inlet had a significant wall thinning due to erosion–corrosion.

**Figure 8:**
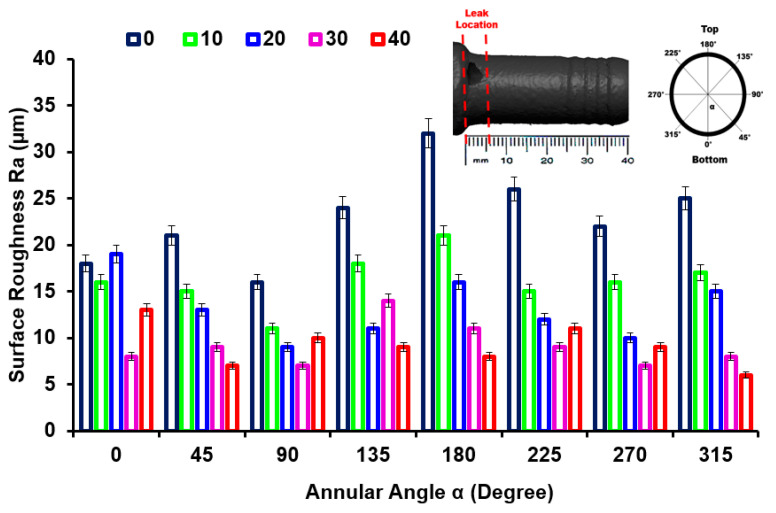
Surface roughness Ra at different locations in the nozzle pipe.

**Figure 9:**
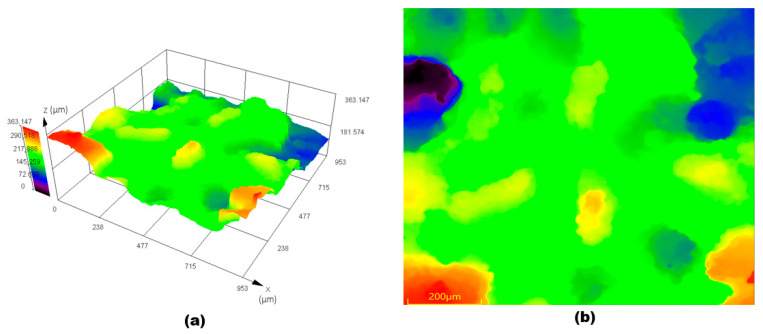
Surface topography of the adjacent surface of the leak location: (**a**) 3D profile; (**b**) 2D profile.

Figure 8 shows the surface roughness Ra variation from inlet to outlet after the removal of the corrosive layer. The surface roughness Ra adjacent to the leak location is 32.1 ± 0.11 μm, which is the minimum surface roughness measured adjacent to the outlet. The decreased roughness from inlet to outlet could be due to less pitting and degradation during erosion–corrosion. It can be seen in Figure 9 that there is an uneven morphology with several peaks and valleys adjacent to the leak location at α = 180° on the x = 0. It can be seen in Figure 9 that there is noticeable surface degradation adjacent to the leak location, leading to the failure of the mild steel pipe nozzle during erosion–corrosion. The results also indicate that the degrading effect and strain-hardening effect on the worn surface adjacent to the inlet enhance wall thinning and cause the failure of the pipe.

### 3.3. CFD-DPM Simulation Results

**Figure 10:**
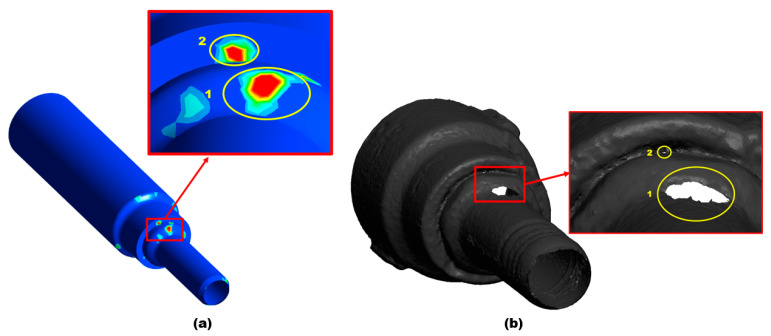
Location of erosion: (**a**) CFD-DPM simulations; (**b**) 3D scan.

The CFD-DPM simulation of the failed jet nozzle pipe is presented in Figure 10. The solid particles in this study are sand particles of 300 µm in size with water carrier fluid, and particle tracking was utilized to identify the maximum degradation zones.

**Figure 11:**
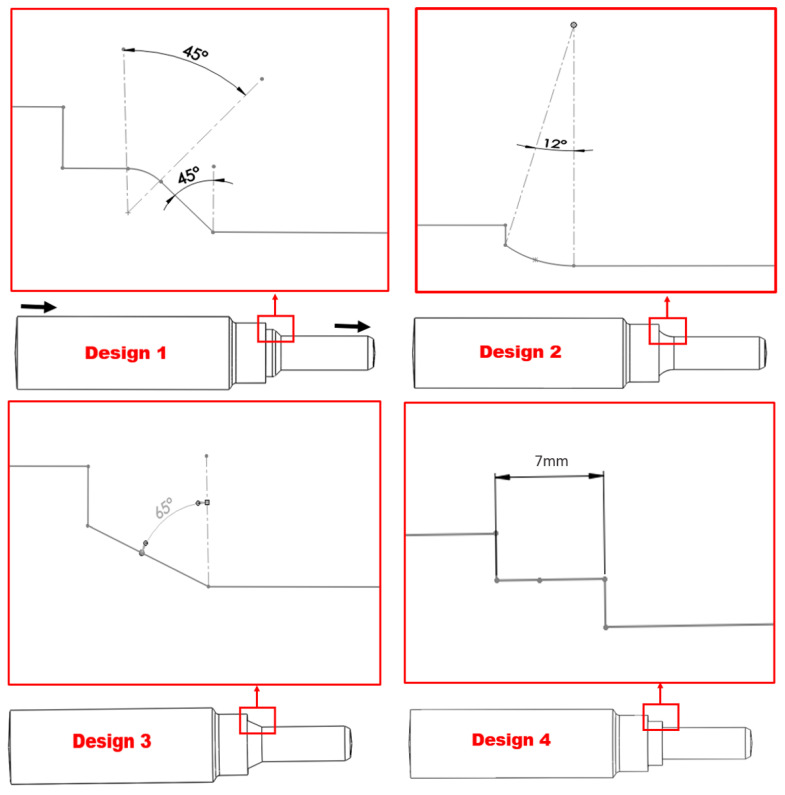
Different design configurations of pipe nozzle.

As determined by the simulation results in Figure 10, the areas highlighted in yellow color, marked locations 1 and 2 on the top of the nozzle pipe, experience the maximum erosion and particle impaction. This indicates that the maximum erosion–corrosion located adjacent to the inlet resulted in leak sites. The corrosion process is accelerated when there are solid particles present in the flow field because they increase the turbulence of the fluid, which in turn enhances the pressure on the outer wall; the maximum erosive zone is located between α = 180° adjacent to the inlet, resulting in a significant loss in the thickness of the wall, whereas at outlet less erosion is observed. To develop an alternative design solution to mitigate erosion near the inlet, CFD-DPM simulations were performed on three different design configurations in this study, as shown in Figure 11. In order to reduce the maximum erosion rate in the nozzle pipe, the reducer section was modified with a fillet radius, chamfer, and rectangular shape as alternative design choices. Design 1 corresponds to a failed nozzle pipe investigated via 3D scanning, thickness loss, SEM-EDS, and surface topography analysis, as detailed in Section 3.1. The choice of alternative design was made after simulating erosive wear in all four designs with similar operating conditions of failed nozzle pipes. In this study, there were four geometrical configurations (reducer section with failed pipe (Design 1); fillet radius (Design 2); chamfer (Design 3); and rectangular shape (Design 4)) as shown in Figure 11. An inlet diameter of 20 mm and an outlet diameter of 10 mm were considered for numerical simulations.

**Figure 12:**
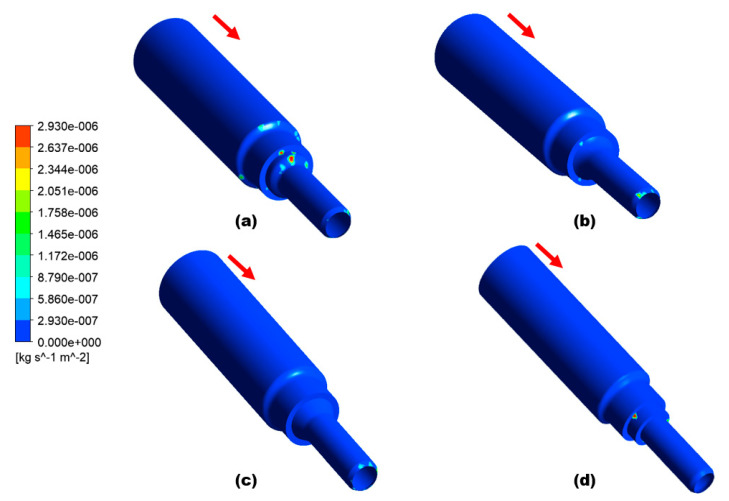
CFD−DPM erosion rate: (**a**) Design 1, (**b**) Design 2, (**c**) Design 3, and (**d**) Design 4.

This simulation analysis shows that particle impaction and turbulence are responsible for the development of the erosion pattern adjacent to the inlet that resulted in the development of leak perforation. In Design 3, the maximum erosion rate is five times less than in Design 1, which indicates Design 3 can be used as a choice of replacement to mitigate the erosion of the nozzle pipe, as shown in Figure 12. In terms of erosive wear, the particle impact energy is strongly related to the velocity and turbulence intensity [2].

**Figure 13:**
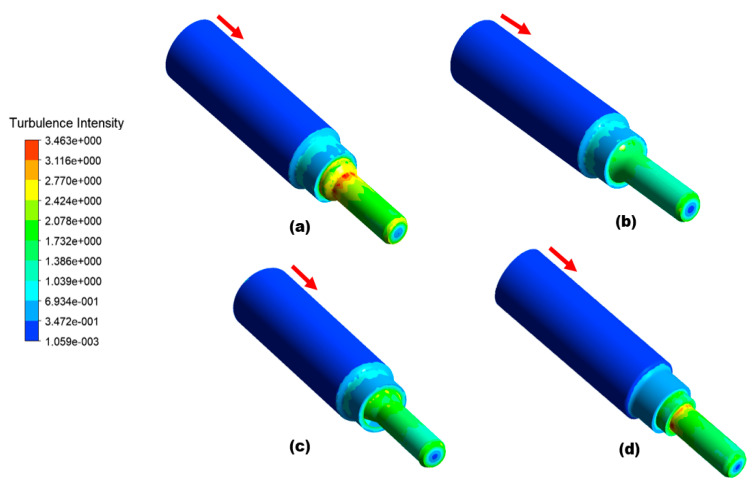
Turbulence intensity: (**a**) Design 1, (**b**) Design 2, (**c**) Design 3, and (**d**) Design 4.

**Figure 14:**
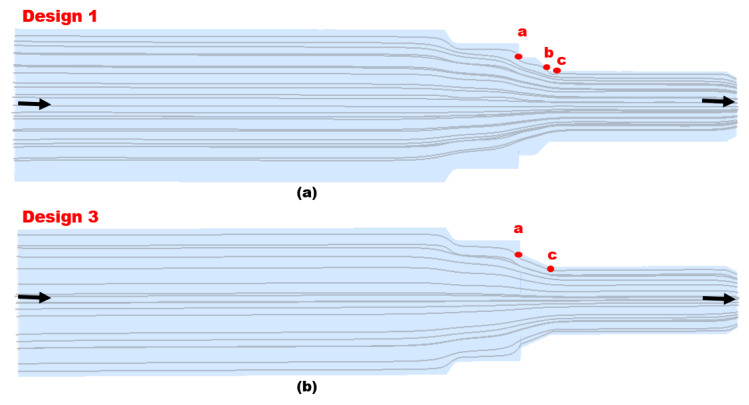
Particle track: (**a**) Design 1 and (**b**) Design 3.

Additionally, the increase in turbulence in Design 1, as shown in Figure 13, escalates particle wall impaction and results in particle wall interaction at locations a, b, and c, as indicated in Figure 14a; it also increases the erosion–corrosion rate adjacent to the inlet. The turbulence intensity in Design 3 is significantly less than in Design 1, causing particle wall impaction near the inlet at locations a and c, as shown in Figure 14b. Under identical operating conditions, Design 3 exhibits a reduction in both turbulence and maximum erosion rate in the area with leak perforations. Consequently, appropriately changing the reducer section of nozzle pipe with chamfer can reduce the maximum particle wall impaction, as shown in Figure 14. The CFD-DPM analysis of all geometric configurations shows that alteration of the design has the greatest impact on erosive wear mitigation. It was found that in mild steel nozzle pipes, the maximum erosion rate at the entry of the pipe gradually increases and results in leak sites and the failure of the nozzle pipe; meanwhile, a recent study has found that in reducer pipes, due to a lack of redirection in the trajectory of the erodent, maximum impaction with the walls occurs at the reducer inlet [33].

To reduce the maximum erosion rate of the failed nozzle pipe, three geometric configurations are proposed for numerical analysis. Design 1 corresponds to the geometry of the failed nozzle pipe. Design 3, with a chamfer reducer section, was found to be the best choice for the replacement of the failed pipe. It can be found that the CFD-DPM simulations predict the maximum erosion rate locations in failed nozzle pipes with reasonable accuracy. However, to increase the accuracy of simulations, simply spherical solid particles should be replaced by non-spherical solid particles. Moreover, particle–particle interactions should also be considered during numerical simulations.

### 3.4. Root Cause Analysis

The condition of the inlet section at the top of the pipe is more severely degraded, and leak perforations were located in a spherical and circular shape in the direction of flow. Pipe misalignment could be the reason for high turbulence, and could consequently enhance the severity of erosive wear on the internal surface. High turbulence means that the particles impact the wall with high frequency, and continuously move around in an annular pattern due to a larger inertia force. Therefore, high turbulence caused more erosion in Design 1 of the nozzle pipe, which is the main cause of leak failure. Based on the EDX assessment of corrosion products, Fe_2_O_3_ formation on the worn surface results in difficulty in passing through oxygen molecules and an increase in oxygen concentration that may result in pitting corrosion and the development of leaks. The non-uniform corrosion characteristics of mild steel result in crack development at the borders of leak locations.

## 4. Conclusions and Preventive Actions

In the current study, the causes of failure in mild steel pipe jet nozzles were analyzed using visual observation, microscopy analysis, thickness loss assessment, surface roughness measurements, and computational fluid dynamics (CFD) simulation techniques.

1. Microscopic and simulation analyses of nozzle pipes reveal that pitting corrosion, cutting, and wall thinning due to highly turbulent water flow carrying sand particles are the primary causes of pipeline failure.
2. Erosion–corrosion is a progressive martial degradation phenomenon; it is recommended that ultrasonic non-intrusive inspection of nozzle pipes connected to direct impact test rigs is carried out at regular intervals. In addition, increased pipe thickness and the use of alloy materials are recommended.
3. CFD-DPM shows that the flow of the carrier phase with the disperse phase impacts more frequently at the inlet location and results in high turbulence at the inlet of the pipe. The simulation results reveal that the maximum erosion rate in Design 3 is five times less than that in Design 1. Based on numerical analysis, Design 1 can be replaced with Design 3 to mitigate the erosion–corrosion rate and consequently decrease the severity of leak failure on the elbow surface during operation.
4. Simulation results indicate that maximum erosion occurs in designs similar to failed pipe nozzles, while minimum erosion occurs in designs with a chamfer reducer section. CFD-DPM can effectively simulate the erosion zone and turbulence inside nozzle pipe configurations.

It should be pointed out that the current study demonstrates that a nozzle pipe with a chamfer reducer section is useful for erosion mitigation in liquid–solid flow conditions. However, the industrial flow process may involve three-phase flow with highly viscous carrier fluids. It is necessary to confirm the proposed design’s erosive wear performance under multiphase flow conditions. For this purpose, experimental evaluations are needed to validate the findings herein, and the influence of design and flow parameters should be considered in future studies.

## Data Availability

Data are contained within the article.
